# Promoting Mental Health, Physical Activity, and Healthy and Sustainable Dietary Behavior Among Practical Education Students in the Netherlands: Protocol for a Multiphase Participatory Research Study

**DOI:** 10.2196/84723

**Published:** 2026-01-14

**Authors:** Daniëlla van Uden, Annemarie Wagemakers, Margot Peeters, Marloes Kleinjan, Kirsten Verkooijen

**Affiliations:** 1 Health and Society Group Wageningen University & Research Wageningen The Netherlands; 2 Youth Studies Department of Interdisciplinary Social Sciences Utrecht University Utrecht The Netherlands; 3 Organisation of Healthcare and Services HAN University of Applied Sciences Nijmegen The Netherlands

**Keywords:** community-based participatory research, empowerment, adolescent health, health promotion, health inequities, student, secondary schools, vocational education

## Abstract

**Background:**

While a healthy lifestyle at a young age benefits youth now and later in life, not all youth have equal access to resources and support for adopting a healthy lifestyle. Most youth health promotion programs target the general adolescent population without addressing underlying equity issues. Similarly, participatory research, a promising methodology for the development of health promotion programs and addressing health equity, leaves youth in more vulnerable positions, often underrepresented. This research addresses these gaps by focusing on participatory research for health promotion program design with youth in practical education (praktijkonderwijs).

**Objective:**

This research has two objectives: (1) to gain insight into how to meaningfully involve youth in vulnerable positions in participatory research for health promotion by focusing on practical education students as a case study, and (2) to gain insight into possible outputs and outcomes of the developed health promotion programs.

**Methods:**

The research in this protocol is part of the LIFTS (Healthy Lifestyle for Low-Literate Teenagers) project, which aims to promote a healthy lifestyle for practical education students using a participatory approach. The current research uses a multiphase mixed methods research design consisting of 3 studies. Regarding the first objective, we conduct a systematic literature review (study 1) and an empirical qualitative study at practical education schools (study 2). The systematic literature review examines current knowledge on empowering approaches to engage practical education students and similar youth groups as coresearchers in health promotion program design and implementation. The qualitative study explores if and how practical education students can meaningfully be engaged as coresearchers in participatory research for health promotion program design. In line with participatory research, we developed research methods in collaboration with practical education schools and relevant LIFTS stakeholders. Regarding the second objective, we will conduct a realist evaluation of the newly designed health promotion programs within LIFTS (study 3).

**Results:**

On April 11, 2025, the protocol for the systematic literature review (study 1) was ready and submitted to PROSPERO. In September 2025, we finished the data collection and analysis of the empirical qualitative study (study 2) and wrote the results. The realist evaluation (study 3) is foreseen for 2026.

**Conclusions:**

This research contributes to the advancement of academic knowledge in the field of health promotion and participatory research with youth in vulnerable positions. We expect to deliver practical recommendations and lessons learned on how to actualize youth empowerment in participatory research for health promotion, which may inspire future researchers. Additionally, we seek to promote health equity in the Netherlands by contributing to the development and implementation of tailored health promotion programs for practical education students.

**International Registered Report Identifier (IRRID):**

DERR1-10.2196/84723

## Introduction

### Background

A healthy lifestyle—comprising regular physical activity, a healthy and sustainable diet, and positive mental health—is widely recognized as a cornerstone of good health across the lifespan. Stimulating a healthy lifestyle at an early age can not only benefit adolescents in the present but also promote the quality of their health in adult life [1-3]. However, not all youth have equal access to a healthy lifestyle, as the ease of adopting and maintaining a healthy lifestyle is strongly affected by social determinants, such as educational level [4]. Secondary education students in vocational tracks generally experience greater challenges to adopt a healthy lifestyle than their peers in academic tracks, both during adolescence and later in life [5]. However, most adolescent health interventions target the general adolescent population, overlooking the role of social determinants shaping health disparities. It is therefore essential to develop and implement youth health promotion programs that cater to underrepresented youth groups to stimulate health equity [6].

Participatory research with youth is a promising methodology for the development of health promotion programs for health equity [7]. In participatory research, scholars strongly collaborate with people with lived experiences by involving them as coresearchers, rather than passive respondents, in various research phases [8]. As a methodology, it prioritizes power-sharing, conducting research with, instead of on, communities, social justice, and locally rooted research approaches [9]. Scholars increasingly value and acknowledge the pivotal impact of participatory research with youth, and a growing number of research projects involve youth as coresearchers [7,10-13]. This trend is commendable, as participatory research with youth can contribute to youth empowerment [13-15], the relevance of interventions for youth [13], the validity of scientific research [13], and health equity [7].

However, it is crucial to recognize the limitations regarding the diversity of youth groups included in participatory research. Current research overrepresents more privileged youth and, therefore, often leaves youth in more vulnerable positions underrepresented [7,16-18]. Vulnerability can be defined based on many characteristics, such as, but not limited to, disability, migration status, and socioeconomic status [19]. Challenges, such as complicated research proposals, fear of rejection by the institutional review board, or fear of inaccessibility of potential participants, can demotivate researchers from conducting research with people in vulnerable positions [20]. Currently, there are still a few examples of studies describing the participatory research process with youth in vulnerable positions to inspire future researchers [21].

Addressing this gap is important, as possibilities for youth empowerment and more equal power relations can be especially valuable for adolescents experiencing greater vulnerability and marginalization [16]. Additionally, participatory approaches provide possibilities to adapt interventions to the needs, wishes, and lived experiences of youth [13]. Therefore, it is precisely the perspective of youth in a vulnerable position that can be crucial for translating general findings to risk populations, for developing appropriate health promotion programs for these groups, and ultimately, for promoting health equity. Furthermore, we lack insight into the outcomes of health promotion programs developed using a participatory approach.

In this research, we aim to address these gaps by focusing on practical education (praktijkonderwijs) students in the Netherlands. In the Dutch context, students enrolled in practical education are often regarded as both vulnerable and underrepresented in participatory research. practical education is a type of Dutch secondary education intended for students aged 12-18 years who are being trained in practical subjects [22]. To our knowledge, practical education is an educational pathway found only in the Netherlands and not easily comparable internationally. Students unable to obtain a diploma in preparatory vocational secondary education (Voorbereidend Middelbaar Beroepsonderwijs [VMBO], EQF level 1-2 [23]) can enroll in practical education [24]. practical education holds two official admission criteria: (1) an IQ between 55 and 80 and (2) a learning delay of 3 years or more in at least two of the following domains: conceptual mathematics, reading comprehension, technical reading, or spelling. The curriculum focuses on personal development and on fostering students’ ability to participate independently in society. After graduation, practical education students generally go on to vocational education or directly enter the labor market [22]. Currently, there are 177 practical education schools in the Netherlands and more than 29,000 practical education students, amounting to about 3% of all students in Dutch secondary education [24]. According to the Sectorraad Praktijkonderwijs [25]—the national body representing and advocating for practical education schools— practical education students are generally in a more vulnerable position due to their limited cognitive capacities in an increasingly complex society, and youth are more often from families with lower socioeconomic status. Because of these vulnerabilities, practical education students may experience more health challenges compared to their peers in other types of secondary schools. Health surveys show, for instance, that practical education students are less physically active [26]. However, there are limited health promotion programs specifically designed for this target group. Tailored programs developed using a participatory approach together with practical education students, their teachers, and caregivers might result in positive changes in the healthy lifestyle of practical education students and ultimately a reduction of health inequities. According to the World Health Organization, health promotion is defined as “the process of enabling people to increase control over and to improve their health” [27]. Actions may focus on individual behavior as well as social and environmental interventions that address the root causes of ill health [27]. As we focus on the school context in this research, we focus on all actions a school takes to promote the health of its students.

### Research Objective and Questions

There is still a limited understanding of how to meaningfully involve practical education students in participatory research for health promotion program design and implementation, as well as of the possible outputs and outcomes of the programs that result from a participatory approach. Therefore, this research aims to shed light on these important issues. The research takes place in a practical education setting with practical education students as the main stakeholders. The two main research questions are as follows:

1. How can practical education students be engaged as coresearchers in health promotion program design and implementation in ways that work empowering?
2. What are the outputs and outcomes of a health promotion program resulting from a participatory research approach in the practical education context, and how are they realized?

## Methods

### Theoretical Framework

The theoretical framework guiding this research is the six-element framework, developed by Chrifou et al [14], and is applied to better understand how to actualize and embed youth empowerment within a participatory research process for health promotion. The authors identify 4 short-term and 2 long-term goals for engaging young people as coresearchers. The short-term goals, which serve to create an enabling environment, include resources, adult facilitation, sense-making, and capacity building. The long-term goals focus on creating opportunities for empowerment and collective participation and include positive child and adolescent development and participatory competence. According to the framework, and in line with Bronfenbrenner’s ecological systems theory [14,28,29], the impact of contextual factors on actualizing youth empowerment is situated within an ecological context, including the macrosystem (society and physical environment), the exosystem (community organizations and institutions), and the microsystem (youth, friends, peers, teachers, and family) [14].

The framework is used in the proposed research to operationalize the concept of empowerment and to synthesize the study results. Specifically, it helps to answer the “how-questions,” namely “How can practical education students be engaged as coresearchers in ways that work empowering?” and “How are the outputs and outcomes of a health promotion program resulting from a participatory research approach in the practical education context realized?”

### Study Design

This research uses a multiphase mixed methods research design consisting of 3 studies. The first study addresses research question 1 through a systematic literature review, while the second study addresses the same research question through an empirical qualitative study. The third study addresses research question 2 and consists of a realist evaluation. During phase 1, we conduct studies 1 and 2 simultaneously to enable an iterative process, which is conducive to answering research question 1. Additionally, using the same analytical framework in these two studies helps us compare the results of both studies and support the iterative process. Phase 2 will consist of study 3.

### Study Setting

#### Overview

The proposed research in this study protocol is part of the LIFTS (Healthy Lifestyle for Low-Literate Teenagers) project (Multimedia Appendix 1), which is funded by the Netherlands Organization for Scientific Research (NWO [Nederlandse Organisatie voor Wetenschappelijk Onderzoek]; grant number KICH1.GZ03.21.011). The main research question of LIFTS is “How to promote, in a participatory way, sustainable, healthy living in practical education students with the use of accessible, acceptable, and engaging technology?” It focuses on 3 health topics, such as mental health, dietary behavior, and physical activity, and covers 4 research phases, such as needs assessment, co-design, implementation, and evaluation. Four PhD researchers are employed at LIFTS, one for each of the three health topics (ie, mental health, dietary behavior, and physical activity), and one PhD researcher to focus on the participatory process across the health topics and research phases. This study protocol involves the aims and activities of the latter PhD researcher.

The LIFTS consortium consists of 3 universities (Wageningen University & Research [WUR], Utrecht University (UU), and Eindhoven University of Technology [TU/e]), practical education schools, industry partners, and societal stakeholders (eg, Knowledge Centre for Sport & Physical Activity Netherlands, Special Heroes the Netherlands, and Trimbos Institute). Three practical education schools host the research activities. To ensure logistical feasibility, we focused on building relationships with the partners to foster collaboration and to ensure logistical feasibility in the preparation phase of LIFTS. In addition, the researchers, their supervision teams, and the consortium as a whole frequently meet to discuss progress and to ensure good alignment between the activities. Furthermore, LIFTS has appointed a program coordinator who acts as a contact person between the partners.

#### Study 1: Systematic Literature Review

We will conduct a systematic literature review to determine the current knowledge on empowering ways to engage practical education students and similar youth groups as coresearchers in health promotion program design and implementation. As practical education is difficult to compare internationally, we will first look into participatory research approaches for health promotion program design and implementation for youth in secondary education in general and how these approaches can be empowering. Next, we will investigate how these empowering participatory approaches differ between educational pathways in secondary education. By distinguishing between educational pathways, we hope to get more insight into empowering participatory research approaches specifically suitable for students in practically oriented or vocational tracks, which can be translatable to the practical education context.

The electronic bibliographic databases that will be used are PubMed, ERIC, and Web of Science. The search language will include Dutch and English, and no date restrictions will be used. The study has been preregistered in PROSPERO (registration number CRD420251010102), where the full search strategy can be found.

Studies will be included in the review if (1) the research takes place in secondary school(s) (including vocational, general, or academic tracks) for youth typically aged 12-18 years; (2) the research activities are participatory in nature for youth involved; and 3) the research activities have the goal to promote health for youth, such as (but not limited to) physical activity, mental well-being, and dietary behavior.

The studies resulting from the search will be screened and selected in 2 steps. In the first step, the reviewers will select the studies by screening the title and abstract against the inclusion and exclusion criteria. In the second step, the full text of the selected studies will be screened against the inclusion and exclusion criteria. Studies found eligible at step 2 will be included in the literature review. We will use the QualSyst checklist developed by Kmet et al [30] to assess the quality of both qualitative and quantitative research included.

To synthesize our data, we will first provide an overview of the different participatory research approaches found in the selected literature. We will do this by creating a table with information about the name of the participatory approach, methods, duration process, ethical considerations, stages of youth involvement, and other outcomes than youth empowerment. Second, we will extract information from the selected studies on how they contribute to youth empowerment based on the elements of the framework of Chrifou et al [14]. Finally, we will examine how the participatory research approaches differ by type of secondary education.

#### Study 2: Qualitative Research

Our second method to explore “how practical education students can be engaged as co-researchers in participatory research for health promotion program design in ways that work empowering” involves an empirical qualitative study. Qualitative research aims to understand and describe people’s lived experiences and worldviews and is therefore most suitable to explore the experiences of youth [31].

### Data Collection

For the second study, we have collected qualitative data among 3 types of stakeholder groups (practical education students, practical education staff members [teachers and management], and researchers) during the needs assessment phase of LIFTS. We gathered information on the preferred, most meaningful, and feasible ways of practical education students’ involvement in the LIFTS research process, as well as the actual experiences of practical education students’ and staff members’ participation in research activities. In line with participatory research, we developed the methods for data collection in collaboration with the relevant stakeholders. We conducted group interviews with practical education students using visual methods, as they are considered suitable for youth [12,32]. We conducted (group and individual) interviews with practical education teachers. The researchers involved in LIFTS maintained a reflection diary during their research activities and took part in a group interview at the end of data collection. Additionally, participant observation was conducted during the participatory research activities taking place at the schools to get more familiar with the practical education school context and the LIFTS research activities and to get to know students and teachers.

### Sampling and Recruitment

The research activities have been designed, planned, and implemented in close collaboration with the staff members of the 3 participating schools. Depending on their expertise, needs, and wishes, the data collection with the practical education students took place (1) at the end of a research activity with the entire class, (2) at the end of a research activity with the class divided into small groups, and (3) at a different moment with a small group of students. Staff members who have been involved in the planning and implementation of research activities were asked to participate in an interview to reflect on their participation. To obtain a balanced and comprehensive answer to the research question, the data collection has covered the health topics and the 3 participating schools as evenly as possible.

### Data Analysis

#### Overview

The interviews with the staff members and researchers were recorded and transcribed, and the data from interviews with students were collected on posters and in field notes. We conducted the data analysis based on the methodology for qualitative data analysis by Beuving and de Vries [33], using open and axial coding. The six-element framework of Chrifou et al [14] serves as an analytical lens to explore whether and how the participatory research process works, empowering in the practical education context.

#### Study 3: Realist Evaluation

To provide future researchers with insights into the working mechanisms of health promotion programs in the practical education context, we will conduct a realist evaluation [34]. Specifically, we will explore the outputs and outcomes of the health promotion program that results from a participatory research approach in the practical education context and understand how these are realized [34]. In line with studies 1 and 2, we will use the framework of Chrifou et al [14] to operationalize empowerment. Realist evaluation is a theory-driven approach to evaluating programs or interventions, focusing on understanding how and why they work (or do not) in specific contexts. Rather than simply measuring outcomes, it explores the underlying mechanisms that produce those outcomes and the conditions that trigger them. It acknowledges that programs may work differently for different people or settings and seeks to identify patterns in these variations. Ultimately, realist evaluation aims to generate transferable lessons by explaining what works, for whom, in what circumstances, and why [35].

Corresponding to a realist approach, we will first develop a program theory. A program theory is a conceptual framework that explains how and why a program is expected to work. It outlines the causal pathways between activities, outputs, and desired outcomes, often including assumptions and contextual factors that influence success. It generally includes so-called context-mechanism-outcome configurations [35], which we also plan to create. Due to the participatory nature of LIFTS, we will keep an open approach toward the data collection. The exact methods for data collection during the test and refinement step depend, among others, on the health promotion program that will be developed. However, we foresee that the program theory and context-mechanism-outcome configurations will be shaped with input from the needs assessment and co-design activities by conducting brainstorming sessions with the 3 LIFTS PhD researchers. The following step in the realist evaluation involves the testing and refinement of the program theory. To do so, we plan to conduct realist interviews [36] with relevant stakeholders (staff, LIFTS partners, and caregivers). When refining the program theory with students, we expect, based on study 2, that a variety of practical, visual, interactive, clearly structured research activities with immediate results work best with practical education students, while quantitative methods (eg, questionnaires) are less suitable.

### Data Triangulation and Integration

Our study promotes data triangulation and integration in several ways. Both studies 1 and 2 serve to answer research question 1 (How can practical education students be engaged as coresearchers in health promotion program design and implementation in ways that are work empowering?). While the qualitative research seeks to gain insight into the practical education context and provide empirical data on the participatory process, the systematic literature study analyzes participatory research for health promotion in secondary education in the literature, which helps to generalize the findings of the empirical qualitative study. Using the framework of Chrifou et al [14], both studies ensure that findings are comparable. The results of studies 1 and 2 also provide input for the participatory research process of the other three LIFTS PhDs. Additionally, the results of studies 1 and 2 inform the methodological design of the realist evaluation, as described in study 3.

### Ethical Considerations

#### Human Subject Ethics Review Approvals or Exemptions

Study 1, the systematic literature study, does not require ethical approval since it does not involve humans. Study 2, the qualitative study, has been approved by the Wageningen University & Research Research Ethics Committee (WUR-REC; approval number 2024-011). Ethical approval for study 3, the realist evaluation, will be sought in due course.

#### Informed Consent

All study participants, teachers, and students are fully informed about the aims of the research prior to participation. Informed consent is obtained from all participants in interviews. Before starting the interview, the researcher goes through the information in the information sheet with participants to make sure that participants understand the aims of the study, know what is expected from them, and know what their rights are. With regard to practical education students, who do not have a high literacy and may not have a signature, we believe an oral informed consent is more suitable for this research. Additionally, passive informed consent is asked from one caregiver by email, in which they are informed about the research, and they have the possibility to opt out by responding to this email. Passive consent has been chosen after consultation with the participating practical education schools and the WUR-REC. We believe passive consent is suitable because there is minimal burden and no risk involved in the interviews with their child. Also, when asking for active informed consent by email, there might be difficulties for caregivers to digitally sign an informed consent form, and they might not respond, which leads to a small pool of students able to participate in this research. Furthermore, active consent could lead to nonparticipation—not because of disagreement with the study—but rather because of forgetting to fill out forms. This may also lead to the exclusion of students who would like to participate and share their voices. After carefully weighing the advantages and disadvantages, the WUR-REC approved this option.

#### Privacy and Confidentiality

LIFTS involves a collaboration between three universities. Four PhD students, who are hosted at these universities (two at WUR, one at UU, and one at TU/e), are the principal researchers of LIFTS. Each PhD student has access to her own data collection, which is stored at a safe (password-protected) place that is also accessible to the supervisors who work in the same research group. The anonymized data are also accessible upon reasonable request by the other PhD students and supervisors in LIFTS. A data management plan, describing how LIFTS data are shared and stored, is in place.

Results from LIFTS will be shared and discussed with the other LIFTS partners (practical education schools, industry partners, and societal stakeholders). However, these partners do not have access to the collected data. A consortium agreement in which the roles and responsibilities of the LIFTS parties are described is signed by all LIFTS partners.

Data are collected, processed, and stored according to the LIFTS data management plan that has been developed according to the WUR privacy policy and regulations. The research described in this protocol neither aims to collect data on confidential or sensitive issues nor to collect special categories of personal data. However, confidential or sensitive issues may be mentioned by the participants. To guarantee confidentiality, names and other information that can be used to trace back to individuals will not be used in the transcription of interviews or observation notes, and audio recordings will be permanently deleted once transcription is complete. Data will be saved in a file only accessible by the researchers involved in LIFTS.

#### Compensation Details

It is important to properly value the youth’s participation and time investment in this research project. However, it is still contested what a suitable (monetary) remuneration is for youth participation [15]. To determine a suitable remuneration for practical education students involved, we will consult the recommendations of the Netherlands Organization for Health Research and Development (ZonMw; ZorgOnderzoek Nederland / Medische Wetenschappen [37]) and determine it together with practical education staff, practical education students, and other LIFTS researchers.

## Results

In March 2023, funding approval for LIFTS was received, and in October 2023, the 5-year project officially started. In January 2024, the author DvU of the studies in this protocol was employed and started to familiarize herself with the practical education context, the project's aims, and the procedures for ethical approval. In March 2024, ethical approval was obtained for the qualitative research (study 2). Data collection for study 2 was started in September 2024 (the beginning of the school year) and ended in January 2025. In total, 179 practical education students participated in the data collection (23 at School A, 91 at School B, and 65 at School C) as part of the needs assessment. Additionally, 17 practical education staff members have been interviewed (3 at School A, 8 at School B, and 6 at School C). Researchers kept a reflection diary during their data collection (from September to January), and in December 2024, the interview was held among the 4 PhD researchers involved in LIFTS. To get more familiar with the practical education school context and the LIFTS research activities and to get to know students and teachers, the author DvU conducted 11.5 hours of participant observation during the participatory research activities taking place at the schools. We have finalized the analysis and are currently, that is November 2025, writing an academic paper about this study. By the end of 2025, the researchers expect to wrap up the results of the needs assessment. The results of this study will also be shared with the LIFTS stakeholders in meetings and the newsletter and will inform the co-design of the health promotion programs of the other three PhDs.

On April 11, 2025, the protocol for the systematic literature review (study 1) was ready and submitted to PROSPERO. The search and selection of studies has started, and it is expected that the systematic literature review will be finished halfway through 2026.

The realist evaluation (study 3) is planned for the school year 2026-2027, when the other PhDs have co-designed an initial design of a health promotion program. At the moment, the other three PhDs are conducting the first cocreation sessions with the practical education schools on the design of the health promotion programs. We expect that they will have the first results in early 2026, and we will design the best evaluation strategy with their input.

## Discussion

### Anticipated Results

This research has a two-fold aim: (1) to gain insight into how to meaningfully involve youth in vulnerable positions in participatory research for health promotion by focusing on practical education students as a case study and (2) to gain insight into possible outputs and outcomes that result from a participatory approach. Given the limited understanding of these issues to date, this research contributes to the advancement of academic knowledge in the field of health promotion and participatory research with youth in vulnerable positions. We expect to deliver practical recommendations and lessons learned on how to actualize youth empowerment in participatory research for health promotion, which should inspire future researchers.

Additionally, we use the six-element framework for actualizing child and adolescent empowerment in participatory action research for health promotion, developed by Chrifou et al [14], as an analytical framework. This framework seems suitable as a theoretical framework for this research because it (1) allows us to operationalize and analyze the ambiguous concept of youth empowerment in the field of participatory research with youth for health promotion, which is the focus of this research; (2) takes context into account, including the social environment of youth; and (3) builds upon previous models conceptualizing participatory research with youth [17,38,39]. The framework has been published quite recently (2024) and, to our best knowledge, has not been practically assessed yet. This research could potentially add to this theoretical framework.

Next to its academic relevance, this research seeks to have a strong societal impact. First, this research aims to contribute to the health and well-being of practical education students. While practical education students show less healthy behavior than their peers in other types of Dutch secondary education [26], there are limited health promotion programs specifically designed for this target group. LIFTS aims to promote a healthy lifestyle among practical education students by developing and implementing health promotion programs on healthy and sustainable dietary behavior, physical activity, and mental health using a participatory approach. This research will contribute to this development by gaining insight into an empowering and meaningful participatory research approach in the practical education context. It explicitly allows moments for reflection and colearning—important aspects of participatory research [8]—that can guide further steps in the participatory process in LIFTS and, consequently, contribute to better health promotion programs for practical education students. In the long term, this research will hopefully contribute to a healthier lifestyle for practical education students and close the gap between practical education students and their peers in terms of health inequities. Second, young people are considered to have the right to influence decision-making and make possible contributions to change in processes that influence their lives [12,40,41]. One important possible benefit of youth participation in research is youth empowerment [14]. As practical education students are less represented than their peers in other types of secondary education in the Netherlands, this research is expected to contribute to youth empowerment among practical education students through participatory research. Finally, practical education is still relatively unknown as a type of secondary education in the Netherlands. This is a missed opportunity, as the labor market needs many practically trained people [42]. Practical education trains students for professions in sectors with high demand in the Netherlands, such as construction, metalwork, landscaping, retail, hospitality, logistics, and health care [22]. As this research focuses specifically on practical education, it contributes to awareness raising and improved image about practical education, not only among the LIFTS stakeholders with whom we work directly but also among an academic audience when presenting the results of this study in papers and at conferences.

### Strengths and Limitations

The first limitation of this research is its context-specific setting. This research is contextualized in an educational pathway in the Dutch education system. As education systems differ considerably between countries, it might be difficult to generalize results directly to different contexts. However, participatory research inherently prioritizes bottom-up and locally rooted research approaches and close collaboration with the community in a colearning process [9]. Generalization is therefore not the main goal of this research. A strength is that we strive to provide an in-depth case study of the practical education context by collaborating with multiple practical education schools, knowledge institutes, and societal and industrial partners. The lessons learned from this research can inspire other researchers and stakeholders working in similar contexts. Nonetheless, the systematic literature review (study 1) helps us to link the empirical results of studies 2 and 3 to the academic literature.

The second limitation is the anticipated low participation of caregivers in the research. As students with learning difficulties seem to experience more social challenges [43], indicating that a good social network seems to be especially important for this group, it is crucial to involve practical education students’ caregivers and teachers in the participatory research process. Until now, we have noticed that it is difficult to involve caregivers, and we expect this might be a challenge throughout LIFTS. To encourage caregivers’ participation in participatory research, the literature emphasizes the need for offering practical opportunities to do so, such as evening focus groups for working caregivers and the provision of childcare or meals [44]. Regarding informed consent, research highlights the importance of providing sufficient information to caregivers, as adults can prevent youth from participating [12]. The strong collaboration with schools and societal partners is a strength, as it can foster recruitment of caregivers in LIFTS and limit the risk of missing an important social environment for practical education students in the design of health promotion programs. Specifically, collaborating with the schools’ community liaisons (brugfunctionaris in Dutch) is an opportunity, as they bridge the gap between schools and caregivers.

### Comparison With Prior Work

To our knowledge, there has been no prior research focusing on participatory research for health promotion in the practical education context. However, participatory research for health promotion has been conducted with somewhat similar youth groups. Prior research exists with VMBO, which, similar to practical education, has a more practical-oriented focus than other educational pathways. For instance, Boonekamp and colleagues [45] have conducted participatory research for physical activity promotion among VMBO students. One of their conclusions is that the development of school-based physical activity programs drawing on active student involvement allows students to shape their physical activity, and it enhances their motivation to engage in physical activity. Another example is the research project VMBO Sportlab [46], which studies how to sustainably enhance the physical activity promotion of VMBO students together with researchers, societal partners, and youth themselves.

An example of a similar methodology to the present research is described by Maenhout and colleagues [21]. The authors share lessons learned from the cocreation of an intervention for physical activity promotion with youth with intellectual disabilities in Belgium. One of the main differences is that the authors rely on researcher reflections, while we also include youth, staff member, and researcher perspectives in study 2. The authors argue that there are still few examples of lessons learned from the participatory research process with youth for health promotion to inspire future researchers. This research starts from the same premise.

### Conclusions

No prior participatory research has been conducted with practical education students for health promotion program design and implementation. The development of tailored health promotion programs for this target group is important to contribute to health equity in the Netherlands. Participatory research is a promising methodology, but we lack knowledge on how to do so in a meaningful and empowering way, as well as of the possible outputs and outcomes of the programs that result from a participatory approach. This research aims to shed light on these issues, contributing to both the academic literature on health promotion and participatory research with youth in vulnerable positions as well as promoting health equity in the Netherlands.

## Appendix

Multimedia Appendix 1 Peer review report from the Netherlands Organization for Scientific Research (NWO).

## Abbreviations

LIFTS: Healthy Lifestyle for Low-Literate teenagers
NWO: Nederlandse Organisatie voor Wetenschappelijk Onderzoek
TU/e: Eindhoven University of Technology
VMBO: Voorbereidend Middelbaar Beroepsonderwijs
UU: Utrecht University
WUR: Wageningen University and Research
WUR-REC: Wageningen University & Research Research Ethics Committee
ZonMw: ZorgOnderzoek Nederland / Medische Wetenschappen
