# Supplementary material for: Promoting Mental Health, Physical Activity, and Healthy and Sustainable Dietary Behavior Among Practical Education Students in the Netherlands: Protocol for a Multiphase Participatory Research Study
Source: JMIR Res Protoc. 2026 Jan 14;15:e84723. doi: 10.2196/84723 (PMC12853082; doi:10.2196/84723)
Supplement: Multimedia Appendix 1 [file resprot_v15i1e84723_app1.pdf]

BIJLAGE: BEOORDELINGSRAPPORT

Financiering: KIC Missie call Zorg in eigen leefomgeving
Dossiernummer: KICH1.GZ03.21.011
Aanvrager: Dr. K.T. Verkooijen
Titel project: LIFTS: Healthy Lifestyle for low liTerate teenagerS

Hoe is de beoordeling van uw aanvraag tot stand gekomen?
De beoordelingscommissie heeft uw bovenvermelde aanvraag, gelijk aan alle andere aanvragen, beoordeeld volgens de procedure en de criteria uit de Call for proposals. Per criterium is uw aanvraag voorzien van een cijfermatige score. Vervolgens heeft de beoordelingscommissie een advies opgesteld. Op basis van het advies heeft het bestuur tot toewijzing of afwijzing van aanvragen besloten.

NWO hanteert in deze ronde de onderstaande scoreschaal en de bijbehorende vertaalslag naar kwalificaties. Een aanvraag moet ten minste de kwalificatie ‘zeer goed’ hebben, alsmede per criterium ten minste de score 4,0 om in aanmerking te kunnen komen voor toewijzing.

Score	Kwalificatie
1,0 t/m 1,4	Excellent
1,5 t/m 3,4	Zeer goed
3,5 t/m 5,4	Goed
5,5 t/m 9,0	Ontoereikend

Uw aanvraag heeft de in onderstaande tabel genormaliseerde gewogen eindscore en kwalificatie ontvangen.

De gegeven scores hebben geleid tot een kwalitatieve prioritering van de aanvragen. In totaal heeft NWO de 6 hoogst geprioriteerde aanvragen van de in totaal 13 ingediende uitgewerkte aanvragen.

De inhoudelijke beoordeling van uw aanvraag
Hieronder vindt u per beoordelingscriterium een uiteenzetting van het oordeel van de beoordelingscommissie.

Beoordelingscriteria	
1. Probleemstelling en –analyse, bijdrage aan oplossing (weging 25%)	1,17
2. Probleemstelling en –analyse, bijdrage aan oplossing (weging 25%)	1,55
3. Kwaliteit consortium (weging 30%)	1,23
4. Kwaliteit onderzoek (weging 20%)	2,28
Eindscore	1,51
Kwalificatie	Zeer goed
Plaats in de prioritering	1

Motivering van de beoordelingscommissie:

Probleemstelling en analyse bijdrage aan oplossing
Het LIFTS-onderzoek (Healthy LIFestyle for low liTerate teenagerS) heeft een duidelijke maatschappelijke urgentie en richt zich op fysieke, sociale en geestelijke gezondheid van laag geletterde tieners om technologische gezondheidsinterventies op maat te bieden. Het voorstel heeft een duidelijke probleemstelling met kennisvragen die gebaseerd zijn op gedocumenteerde discussies over het probleem. Het voorstel heeft een duidelijke probleemstelling en draagt bij aan de oplossing.

Verwachte impact en route naar impact
Het project richt zich op een groep die in potentie levenslang baat kan hebben bij de interventie. Net als de rest van het voorstel is het traject naar impact goed doordacht en duidelijk gepresenteerd. Bovendien omvat het consortium een grote verscheidenheid aan stakeholders in bijvoorbeeld lichaamsbeweging, gezondheidsbevordering, praktijkonderwijs en kennisvalorisatie en -verspreiding. De commissie heeft haar bedenkingen bij de gevoeligheid van sociale druk onder deze groep, sociale druk kan wellicht een negatief effect hebben voor de interventie op de lange termijn.

Kwaliteit consortium
Het consortium is interdisciplinair samengesteld, met achtergronden en expertise in psychologie, voeding, technologie, publieke gezondheid, sociale wetenschappen en gedragswetenschappen. Uit het voorstel wordt de toegevoegde waarde van de samenwerking duidelijk. De directe betrokkenheid van meerdere praktijkonderwijsscholen bij het project wordt door de commissie gewaardeerd; dit verhoogt de kans dat de projectbevindingen direct bruikbaar zullen zijn.

Kwaliteit onderzoek
De commissie is van mening dat het voorstel haalbaar en passend is, gesterkt door de compleetheid van het consortium en de financiële keuzes. De belangrijkste risico's van het project zijn door de onderzoekers geïdentificeerd. Bovendien zijn de werkpakketten volgens de commissie zeer geschikt voor het behalen van de doelstellingen van het voorstel. De commissie is niet volledig overtuigd dat er voldoende studenten en verzorgers betrokken zullen zijn bij het pilottesten op effectiviteit, aangezien daar maar een kleine vergoeding tegenover staat.
